# Clinical effectiveness of aloe vera gel as an adjunct to mechanical debridement in patients with periodontitis: A systematic review and meta-analysis

**DOI:** 10.34172/japid.2025.002

**Published:** 2025-01-20

**Authors:** Nisha Ashifa, Krishnan Viswanathan, Sivapragasam Srinivasan, Varsha K. Pavithran, Shiva Shankar, Rajasekar Sundaram, Senthil Kumar, Divvi Anusha

**Affiliations:** ^1^Department of Periodontology, Rajah Muthiah Dental College & Hospital, Annamalai University, Annamalai Nagar, Tamil Nadu, India; ^2^Public Health Researcher & Biostatistician, Rypple Foundation Cypresstraat 64, 2565LW Den Haag, India; ^3^Department of Public Health Dentistry, Indira Gandhi Institute of Dental Sciences, Sri Balaji Vidyapeeth, Puducherry, India

**Keywords:** Aloe vera gel, Dental scaling, Meta-analysis, Periodontitis, Root planing, Systematic review

## Abstract

**Background.:**

This study aimed to evaluate the clinical effect of aloe vera gel as an adjunct to scaling and root planing (SRP) on plaque index (PI), gingival index (GI), and probing pocket depth (PPD) in periodontitis treatment.

**Methods.:**

Randomized controlled trials (RCTs) were included, focusing on aloe vera gel as an adjunct to SRP in periodontitis patients. An extensive literature search was conducted across PubMed, PubMed Central, Scopus, OVID, Google Scholar, and Cochrane Library databases up to October 2024. The risk of bias was assessed using the Revised Cochrane Risk of Bias tool (ROB 2.0), and statistical analysis was performed using Review Manager 5.4.1.

**Results.:**

Fifteen RCTs were included in this systematic review, and separate meta-analyses were conducted for each outcome. For PI, the random effects model showed a mean difference (MD) of -0.23 (95% CI: -0.69, 0.23), favoring the experimental group (*P*=0.33). The fixed effects model for PI favored the control group (MD=0.12; *P*=0.20). For GI, the random effects model favored the experimental group (MD=-0.28, *P*=0.01), while the fixed effects model favored the control group (MD=0.17; *P*<0.001). For PPD, the random effects model favored the experimental group (MD=-0.45, *P*=0.009).

**Conclusion.:**

As an adjunct to SRP, aloe vera gel significantly improved PI, GI, and PPD in periodontitis patients.

**Trial registration.:**

PROSPERO ID: CRD42020201315.

## Introduction

 Periodontitis is a globally prevalent oral disease affecting the periodontium. It presents with gingival inflammation, connective tissue degradation, and alveolar bone loss, eventually leading to tooth loss. The complex interaction between the pathobionts and the host immune response is responsible for the disease’s commencement and progression.^1,2^

 Periodontal treatment is primarily concerned with reducing the burden of pathogenic subgingival bacteria and resolving inflammation.^2-4^ The initial step in periodontal treatment is the nonsurgical management, which consists of mechanical debridement, supra- and sub-gingival irrigation, and the use of additional chemotherapeutic agents.^2,3^ Mechanical debridement refers to scaling and root planing (SRP), which is the elimination of supragingival and subgingival deposits using hand and/or power-driven instruments. Thus, SRP helps restore periodontal health by reducing microbial load.^2,5,6^

 Various treatment options are being used secondary to SRP, including systemic and locally applied antimicrobial agents, antiseptics, anti-inflammatory agents, and nonsurgical use of lasers.^5,7^ These adjunctive aids enhance the effects of SRP and promote healing. Systemic and local antimicrobial agents have been widely used. However, these agents are associated with adverse effects like antimicrobial resistance, gastrointestinal intolerance, altered taste sensation, staining of teeth, and many more.^8-11^ Recently, phytotherapeutic agents have garnered significant attention as supplementary treatments to nonsurgical periodontal therapy due to their positive qualities like availability, good patient tolerance, reduced cost, and reduced side effects.^8,12^

 Aloe vera, botanically known as *Aloe barbadensis*, is a highly valued medicinal plant from the family *Liliaceae*. It is widely recognized for its wound healing, anti-inflammatory, immunomodulatory, antimicrobial, and antioxidant properties. It also stimulates epithelial cell migration and collagen maturation, promoting tissue regeneration.^13,14^ It is used to treat wounds, burns, skin disorders, infections, gastrointestinal disorders, hemorrhoids, hair loss, and sinusitis.^13,15,16^ In dentistry, aloe vera is used as a topical applicant for oral lesions like aphthous ulcers, oral lichen planus, pemphigus, angular cheilitis, herpetic lesions, oral submucous fibrosis, traumatized tissues, on extraction sockets, chemical burns, in denture stomatitis and periodontally infected sites.^13,17^

 The pharmacological qualities of aloe vera make it valuable for the treatment of periodontitis.^13,14,17^ When used as a mouthwash, aloe vera exhibits optimistic results in resolving gingival inflammation, with no documented side effects.^15^ In patients with periodontitis, subgingival placement of aloe vera gel or chip significantly improved clinical parameters.^5,13,18-20^ Singh et al.^20^ found a significantly higher level of antioxidant levels in GCF after applying aloe vera gel to periodontitis patients.

 In today’s world, clinicians are expected to keep pace with advancements in knowledge and clinical practice. Evidence-based practice is the application of research findings into clinical practice. Considering the usefulness of aloe vera in treating periodontal diseases, the present systematic review/meta-analysis addresses the question, “What is the effectiveness of aloe vera gel used as an adjunct to mechanical debridement in the treatment of periodontitis, when compared to SRP alone or with placebo?”

## Methods

###  Registration and protocol

 The study protocol was registered at PROSPERO International Prospective Register of Systematic Reviews (ID: CRD42020201315). This systematic review and meta-analysis were prepared in accordance with Preferred Reporting Items for Systematic Reviews and Meta-analyses (PRISMA) guidelines^21^ and the Cochrane Handbook of Systematic Reviews and Interventions.^22^

###  Focused question

 The present study focuses on the question, “What is the effectiveness of aloe vera gel used as an adjunct to SRP in the treatment of periodontitis when compared to SRP alone or with a placebo?”

###  Eligibility criteria

 A PICO-based search strategy was developed as follows.

###  Study characteristics in PICO format

 Randomized controlled trials (RCTs) were included in this study.

 Participants (P): Patients with periodontitis with a probing pocket depth (PPD) of 4‒8 mm

 Intervention (I): Subgingival administration of aloe vera gel as an adjunct to SRP

 Comparison (C): With SRP alone or with a placebo

 Outcome (O): Plaque index (PI), gingival index (GI), PPD

###  Inclusion criteria

1. RCTs done on patients with periodontitis with a PPD of 4‒8 mm
2. RCTs done with aloe vera gel as an adjunct to SRP with 4‒6 weeks of follow-up
3. No restrictions on age and gender
4. Full-text articles
5. RCTs published until October 2024
6. Articles published in the English language

###  Exclusion criteria

1. Studies using aloe vera in any other form were not considered.
2. Non-randomized trials (controlled/uncontrolled), case series, case reports, descriptive and analytical studies, in vitro studies, animal studies, review papers, letters to the editor, monographs, and conference papers were excluded.
3. Literature in other languages that could not be translated by the reviewer was excluded.
4. Unpublished data with full access.

###  Information sources

 An extensive electronic search was conducted on PubMed/MEDLINE, PubMed Central, OVID, Google Scholar, and Cochrane Library databases up to October 2024 to determine the eligible studies for this review. Further studies were found by hand-searching the reference lists of the selected papers.

###  Search terms used ( MeSH terms)

 The search terms used to identify relevant articles were periodontitis, adult periodontitis, chronic periodontitis, aloe vera gel, aloe, scaling, dental, supragingival, supragingival scaling and root scaling, root planing, planing, nonsurgical periodontal therapy, local drug therapy, local drug delivery, local drug delivery (LDD), outcome, treatment, patient-relevant outcome, clinical effectiveness, treatment effectiveness, rehabilitation outcome, outcome, rehabilitation, effectiveness.

###  Study selection process

 Two reviewers (NA and KV) independently examined the titles and abstracts of all studies during the preliminary round of study selection. A third author (RS) was brought in to settle the differences over the eligibility of the articles. Irrelevant studies were excluded. The full texts of the articles that met the inclusion criteria and the keywords were gathered. Further screening of the full texts of the selected articles was performed during the second round of the study selection. Articles that did not match the inclusion criteria were excluded from consideration, and the reasons for exclusion were noted (Table 1).

###  Data collection process and data items

 Data from the chosen articles were collected by two reviewers (NA and KV) using data extraction forms, which included details like the details of the study, year of publication, study design, participants and grouping, intervention and comparison, number of applications of aloe vera gel, parameters assessed, follow-up, statistical analysis used, and outcome. A third reviewer (SK) settled the disagreements over the data to be extracted.

###  Risk of bias assessment 

 Two reviewers (NA and SS) used the ROB2.0 tool to evaluate the risk of bias in the included RCTs, adhering to the Revised Cochrane Risk of Bias guidelines.^42^ This instrument consists of five items, which include bias due to randomization, deviation from intended intervention, missing outcome data, measurement of the outcome, and selection of the reported result. The articles were deemed low risk if all the criteria were met, high risk if one or more criteria were not met, and some concerns of bias if one or more criteria were partly met or had insufficient information. Finally, the overall bias for each article was also assessed. Disagreements regarding the same were settled by a third reviewer (VKP).

###  Effect measures and synthesis of results

 Data on the outcomes were extracted from each study and initially entered into Microsoft Excel. The statistical analysis was conducted using the licensed Review Manager version 5.4.1 *[Review Manager ( RevMan ) [Computer program]. Version 5.4. The Cochrane Collaboration, 2020.* The chi-squared test and I^2^test were used to calculate the heterogeneity between the studies. An inverse variance statistical method along with random effects analysis model was employed for an expected outcome of continuous data type, and the effects estimate measure was expressed as mean difference (MD) with totals, subtotals, and 95% confidence interval. The pooled effect measure (overall effect) was estimated using the Z test. The level of significance was determined at *P* ≤ 0.05. Forest plots and funnel plots were created for graphical presentations of results.

## Results

###  Study selection

**Figure 1:**
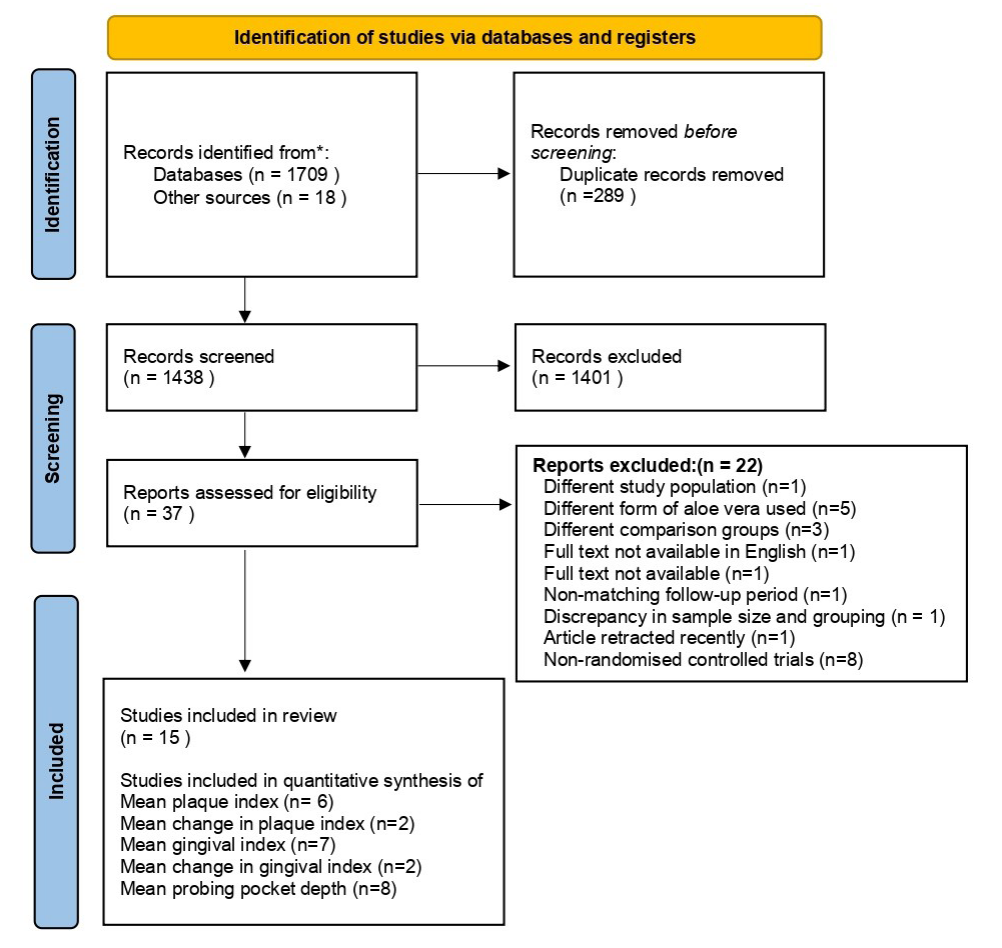
PRISMA flow diagram 2020. PRISMA: Preferred Reporting Items for Systematic Reviews and Meta-analysis

 A total of 1727 articles were found in the search results (1709 from databases and 18 from additional sources). After removing duplicates, 1438 articles were screened for the titles and abstracts. Following screening, 1401 articles were eliminated, and 37 articles were retained. The full texts of these 37 articles were reviewed, and 22 articles were excluded. Finally, 15 articles were included in the systematic review.^1,5,18,20,43-53^ Out of these 15 articles, six articles^18,45,48,50,51,53^ were used for the meta-analysis of mean PI, two^49,51^ were used for meta-analysis of mean change in PI, seven articles^18,45,46,48,50,51,53^ were used for the meta-analysis of mean GI, two^49,51^ were used for meta-analysis of mean change in GI, and eight articles^18,20,45,46,48,50,51,53^ were used for meta-analysis of PPD (Figure 1). An overview of the articles that were excluded is provided in Table 1.

###  Study characteristics

 This study analyzed 15 RCTs.^1,5,49–53,18,20,43-48^ All these studies evaluated the clinical efficacy of subgingival placement of aloe vera gel as an adjunct to SRP in patients with periodontitis. Participants in the test group received SRP + aloe vera gel, whereas the control group received SRP or SRP plus a placebo gel. Table 2 presents the characteristics of the included studies. Out of 15 trials that were included, 11 trials reported a single application of aloe vera following SRP,^1,5,53,18,20,43,45-47,51,52^ while four reported multiple applications.^44,48-50^ Two RCTs used Curagel (by Cure Pharma),^48,49^ two RCTs used 98% aloe vera gel,^5,18^ one RCT used 2.5% aloe vera gel,^51^ and one RCT used 99% aloe vera gel.^45^ The follow-up time in all trials ranged from 3 weeks to 12 months.

###  Risk of bias assessment

**Figure 2:**
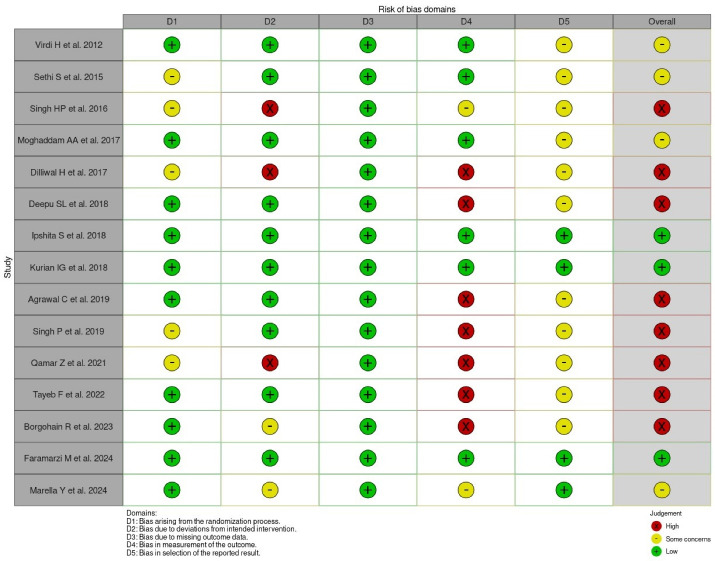
Traffic-light plot of ROB 2.0 assessment of the included studies

**Figure 3:**
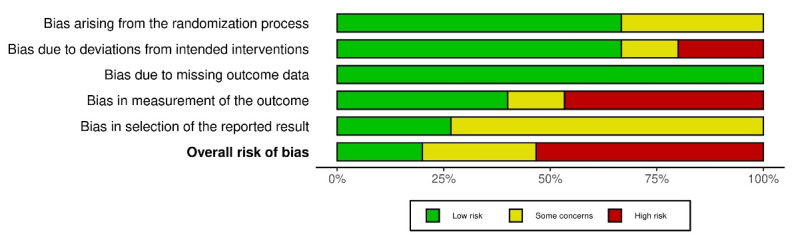
Summary bar plot of ROB 2.0 assessment of the included studies

 The graphs for ROB2.0 were generated using *Robvis.*^54^The domain-level judgments for each study in the Traffic light plot (Figure 2) showed that three studies had a low overall risk of bias, four studies had some concerns, and eight studies had a high overall risk of bias. The distribution of risk-of-bias judgments within each bias domain in the Summary bar plot (Figure 3) depicted a 20% low bias, 27% unclear bias, and 53% high overall risk of bias. The lowest risk was observed in the three main areas of conducting an RCT – missing outcome data (100%), deviations from intended interventions (67%), and randomization process (67%), thus assuring the strength of the methodology of the included studies. However, the highest risk was observed in the measurement outcome (48%) due to the inconsistencies in the measurement of the outcome variable using other indices and non-blinding of investigators

###  Meta-analysis of PI 

####  Mean PI (random effects model)

**Figure 4:**
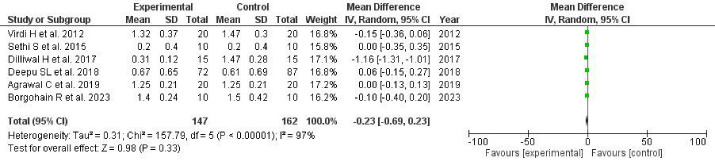
Forest plot of the mean plaque index

 The pooled MD in a random model analysis of PI on teeth at 4‒6 weeks post-intervention was -0.23 (pooled 95% CI: -0.69, 0.23; *P* = 0.33), favoring the experimental group. Out of the total 6 studies included in the meta-analysis of PI, three studies^45,48,50^ showed MD of PI favoring the experimental group (aloe vera + SRP), two studies^18,53^ showed no MD, and one study^51^ favored the control group (SRP alone), all with narrower 95% CI. The treatment (aloe vera adjunct to mechanical debridement) in periodontitis patients had a 50% effect on reducing PI scores in 4‒6 weeks. The I^2^ statistic of PI on teeth post-intervention showed significant heterogeneity of 97% (Tau^2^ = 0.31; χ^2^ = 157.79, df = 5, *P* < 0.00001). The test for overall effect non-significantly favored the experimental group (Z = 0.98; *P* = 0.33) (Figure 4).

####  Mean changes in PI (fixed effects model)

**Figure 5:**
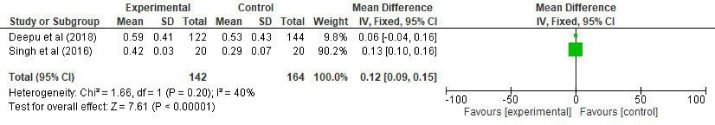
Forest plot of mean changes in plaque index

 The fixed effects model showed an increased PI (MD = 0.12; 95% CI: 0.09, 0.15; *P* = 0.20, not significant) at 1 month, favoring the control group, with the I^2^ statistic showing moderate heterogeneity (40%) (Figure 5).

###  Meta-analysis of GI

####  Mean GI (random effects model)

**Figure 6:**
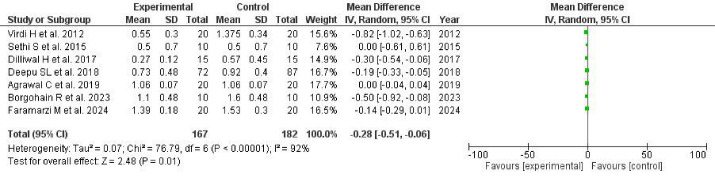
Forest plot of the mean gingival index

 A significant pooled mean difference in the GI of teeth post-intervention (4‒6 weeks) was observed in a random model analysis. The overall effect was -0.28 (pooled 95% CI: -0.51, -0.60), favoring the experimental group (Z = 2.48; *P* = 0.01). Among the 7 studies used for the meta-analysis of the GI, five studies^45,46,48,50,51^ favored the experimental group, and two studies^18,53^ had no MD, with a considerably broader 95% CI compared to the PI analysis. The treatment for periodontitis patients using aloe vera as an adjunct to mechanical debridement showed a 71% effect on reduction in the GI scores in 4‒6 weeks. The heterogeneity test showed the I^2^ statistic of the GI to be significant with 92% heterogeneity (Tau^2^ = 0.07; χ^2^ = 76.79, df = 6, *P* < 0.00001) (Figure 6).

**Table 1:** Excluded studies and the reasons for exclusion

**Author, Year**	**Reason**
Pradeep et al, 2016 ^14^ (n = 1)	Conducted the study in patients with Type 2 diabetes mellitus
Rathod et al, 2015 ^19^ Rithesh K 2021^23^ (n = 2)	Used aloe vera chip as an adjunct to SRP
Choudhary et al, 2020^24^ (n = 1)	Used Aloin (aloe vera extract) as an adjunct to SRP
Gupta et al, 2021^25^ (n = 1)	Implemented subgingival irrigation of aloe vera, and the outcome was compared with chlorhexidine irrigation
Sharma et al. 2018^26^ (n = 1)	The effect of aloe vera gel was compared with probiotic lozenges
Sahgal et al, 2015^27^ (n = 1)	The follow-up period of the study is 7 days
Penmetsa et al, 2019^28^ (n = 1)	The effect of aloe vera as an adjunct was compared with 1% Ornidazole and 0.25% chlorhexidine gluconate gel
Kumar et al, 2015^29^ (n = 1)	The effect of aloe vera gel is compared with propolis gel
Hermanto et al, 2015^30^ (n = 1)	Full text not available in English
Bommireddy et al, 2023^31^ (n = 1)	Full text not available. Abstract published in Special Edition of 2023 FDI World Dental Congress
Sayar et al, 2021^32^ (n = 1)	Used aloe vera toothpaste
Elsadek et al, 2020^33^ (n = 1)	Article retracted recently
Vijay et al, 2022^34^ (n = 1)	Discrepancy in the sample size and grouping
Bhat et al, 2011^13^ Dodwad and Arora 2011^35^ Abdelmonem et al, 2014^36^ Sangwan et al, 2017^37^ Nazir & Kumar, 2018^38^ Phatale & Chavda, 2020^39^ Timothy & Rajasekar, 2020^40^ Katariya & Rajasekar, 2024^41^ (n = 8)	Non-randomized clinical trials

n = Number of studies.

**Table 2:** Characteristics of the studies included in the systematic review

**Study and year**	**Study design**	**Participants & group**	**Intervention & comparison**	**Number of applications of Aloe vera gel**	**Parameters assessed**	**Follow-Up**	**Statistical analysis used**	**Outcome**
Virdi et al, 2012^48^	Split-mouth, randomized controlled study	20 patients divided into test and control group	SRP only + Aloe vera gel vs SRP only	Multiple applications at Baseline 1^st^ week 2^nd^ week	PI GI PPD	6 weeks	Paired t-test, ANOVA	The intragroup comparison revealed a statistically significant difference in PI, GI, and PPD scores. On intergroup comparison, a statistically significant difference was present in the GI and PPD scores but not in PI scores at 6 weeks.
Sethi et al, 2015^18^	Split-mouth, Randomized controlled study	10 patients, each with at least 3 sites in different quadrants, were included.	SRP only + Aloe vera gel vs SRP only	Single application – at baseline	PI GI PPD	3 weeks 6 weeks	paired t-test, one way ANOVA, Post-hoc Turkey’s test, Kruskal Wallis ANOVA, Mann-Whitney test, Fisher’s exact test.	On intra-group analysis, there was a significant reduction (*P* < 0.01) of PI, GI, and PPD & in all the treatment groups. On intergroup analysis, no significant difference was observed across groups at 3 and 6 weeks.
Singh et al, 2016^49^	Split-mouth, Randomized controlled study	60 sites from 20 patients, out of which 40 sites were test sites, and 20 sites were control sites	SRP + Aloe vera gel (Curagel) vs SRP	Multiple applications – at Baseline 7^th^ day	PI GI PPD	30 days 60 days 90 days	Paired t-test, ANOVA	On intergroup comparison, scores of PI and GI were statistically significant (*P* < 0.05), but PPD was not statistically significant (*P* = 0.15)
Moghaddam et al, 2017^5^	Split-mouth, randomized clinical trial	20 patients divided into test and control sites	SRP vs SRP + 98% Aloe vera gel	Single application – at baseline	PI GI PPD	30^th^ day 60^th^ day	Repeated ANOVA measures, independent test, Kolmogrov-Smirnov test	On intergroup comparison, differences in PI, GI, and PPD on the 30th and 60th day between the cases and control group were statistically significant.
Dilliwal et al, 2017^50^	Split-mouth, randomized controlled clinical trial	30 sites from 15 patients divided into 2 groups – Group I and II	SRP + Aloe vera gel vs SRP only	Multiple applications at Baseline 7^th^ day 15^th^ day	PI GI PPD	30 days	Friedman test, Wilcoxon signed-rank test, Mann-Whitney U test	Both intragroup and intergroup comparisons revealed that the mean PI, GI, and PPD were statistically significant on day 30
Deepu et al, 2018^51^	RCT	Total 71 patients divided into 2 groups: Test group -33 Control group -38 Total Sites: 266 Test group: 122 Control Group: 144	SRP + 2.5% Aloe vera gel vs SRP + Placebo gel	Single application – at baseline	PI GI PPD	1 month 2 months 4 months	Independent t-test	A statistically significant difference in PPD and GI was observed in the test group compared to the control group in the 1st month but not in 2nd and 4th month. However, a significant difference in PI was noted for both groups in the 2nd month but not in 1 and 4 months.
Ipshita et al, 2018^1^	Single-center, randomized, controlled clinical trial	30 patients in each test and control group	SRP + placebo gel LDD vs SRP + Aloe vera gel LDD	Single application – at baseline	PI PPD	6 months 12 months	Mean ± standard deviation, one-way ANOVA, Scheffe’s post hoc tests, repeated measures ANOVA	The mean PI scores were not statistically significant; however, the mean PPD scores showed a statistically significant difference at 6 months and 12 months
Kurian et al, 2018^52^	Randomized, single-center, longitudinal, parallel-arm design study	30 patients in each test and control group	SRP + placebo gel vs SRP + 1% Aloe vera gel	Single application – at baseline	PI PPD	6 months 12 months	Mean ± standard deviation, one-way ANOVA, Scheffe’s post hoc tests, repeated measures ANOVA	The mean PI scores were not statistically significant, but there was a statistically significant difference in the mean PPD scores at 6 months and 12 months
Agrawal et al, 2019^53^	Split-mouth, randomized controlled study	40 sites from 20 subjects divided into a test group and a control group	SRP + Aloe vera gel vs SRP only	Single application – at baseline	PI GI PPD	1 month	Paired t-test, independent t-test	The intragroup comparison revealed a statistically significant reduction in the mean PI, GI, and PPD 1 month in both groups (P < 0.001). On intergroup comparison, a statistically significant difference in PPD between the groups at 1 month (*P* < 0.05)
Singh et al, 2019^20^	Split-mouth randomized controlled study	30 subjects	SRP + 80% Aloe vera gel vs SRP only	Single application – at baseline	PPD	1month	Student’s t-test, repeated ANOVA	No statistical significance was observed in PPD between the test and control group at baseline and 1-month follow-up
Qamar et al, 2021^43^	Randomized controlled study	50 patients in each test group and control group	SRP vs SRP + Aloe vera gel	Single application – at baseline	PI PPD	3 months 6 months	Kruskal- Wallis test, Bonferroni`s post-hoc test	Statistically significant improvement was noted for PI and PPD in the test group at follow-up, compared to the control group.
Tayeb et al, 2022^44^	RCT	15 patients in the test group and 15 patients in the control group	SRP + Aloe vera gel vs SRP only	Multiple applications – at Baseline 1^st^ week 2^nd^ week	GI PPD	3 months 6 months 9 months	ANOVA test, Friedman test, Bonferroni’s post hoc test	AV group showed a statistically significant reduction in PD compared to the control group at 3, 6, and 9 months. With respect to GI, a statistically significant difference was noted at 6 months and 9 months follow-up, but not at 3 months follow-up.
Borgohain et al, 2023^45^	Split-mouth randomized controlled study	10 patients	SRP vs SRP + 99% Aloe vera gel	Single application – at baseline	PI GI PPD	30days	Not mentioned	The intergroup comparison between the test and the control group for all parameters at baseline and on the 30th day after the intervention revealed statistically significant differences in terms of PPD and GI.
Faramarzi et al, 2024^46^	Double-blind randomized split-mouth study	20 patients	SRP vs SRP + Aloe vera gel	Single application – at baseline	GI PPD	30days	Kolmogorov-Smirnov test, paired t-test, independent t test	A significant difference was noted between the test and the control group at 30 days follow-up
Marella et al, 2024^47^	Single-blind randomized split-mouth study	20 patients	SRP vs SRP + Aloe vera gel	Single application – at baseline	PI PPD	3 months	Mann–Whitney U test, Wilcoxon signed-rank test, chi-squared test, McNemar’s test	The significant differences observed between the test and control groups in PI and PPD parameters at three months, with no notable differences at baseline, confirm the greater beneficial effect of the intervention in the test group.

Abbreviations: ANOVA: analysis of variance, GI: gingival index, PI: plaque index, PPD: probing pocket depth, SRP: scaling and root planing; RCT: Randomized controlled trial.

####  Mean changes in GI (fixed effects model)

 The fixed effects model showed an increased GI (MD: 0.17; 95% CI: 0.12, 0.23; *P* = 0.00001, significant) at 1 month, favoring the control group, with the I^2^ statistic showing zero heterogeneity (0%).

###  Meta-analysis of PPD

####  Mean PPD

 A significant pooled mean difference in a random model analysis of PPD on teeth at 4‒6 weeks post-intervention was -0.45 (pooled 95% CI: -0.78, -0.11), favoring the experimental group (Z = 2.63; *P* = 0.009). Out of the total 8 studies included for the meta-analysis of PPD, seven studies^20,45,46,48,50,51,53^ showed MD of PPD, favoring the experimental group, and one study^18^ favored the control group, all with a broader 95% CI. The use of aloe vera and mechanical debridement in periodontitis patients showed an 88% effect in reducing the PPD levels after 4‒6 weeks. The I^2^ statistic of PPD post-intervention showed a significant heterogeneity of 84% (Tau^2^ = 0.16; χ^2^ = 42.78, df = 7, *P* < 0.00001).

####  Mean change in PPD

 Only one eligible study reported a significant mean change in PPD between the test and control groups; hence, a meta-analysis was not conducted.^51^

###  Publication bias

 A funnel plot was prepared to assess the publication bias in all the studies included in the meta-analysis. The plotting consists of an effect estimate (mean difference) on the X-axis and a standard error of the mean difference on the Y-axis. Each circle represents the individual study effect estimate. Aggregation of individual study effect estimates was observed to coincide with the overall effect estimate line (middle line) and well within the 95% CI of the funnel, representing the majority of the larger precision studies on the top of the funnel as depicted in the GI and no publication bias. The funnel plots of PI and PPD had most of the studies plotted on the middle and lower section of the funnel, suggesting lower precision, indicating no publication bias in the current meta-analysis.

## Discussion

 Aloe vera is a medicinal plant renowned for its diverse therapeutic properties, including promoting wound healing, providing pain relief, reducing inflammation, and exhibiting antibacterial, antifungal, antiviral, antioxidant, and immunomodulatory properties.^55^ It also offers protection against radiation-induced mucositis and lowers the likelihood of oral thrush in patients receiving radiotherapy.^56^ Aloe vera has various applications due to its medicinal properties in periodontics. In 2014, Dhingra, in his systematic review, concluded that, although the studies included reported the aloe vera dentifrices to be equally effective as conventional dentifrices, the outcomes could not be conclusively reported due to the significant risk of bias of the studies.^57^ Another systematic review by Al-Maweri et al^15^ stated that aloe vera mouthrinse was equally effective as chlorhexidine in minimizing gingival inflammation and less effective than chlorhexidine in minimizing plaque. Recently, Jadhav et al^58^ reported that locally delivered aloe vera significantly improved periodontal parameters.

 The current systematic review focuses on the question, “What is the clinical effectiveness of the subgingival application of aloe vera gel as an adjunct to SRP in the treatment of periodontitis?”

 RCTs available on different databases until October 2024 were included in this review, as they are categorized as Level II evidence in the hierarchy. An extensive literature search was conducted, and 15 RCTs that fulfilled the eligibility criteria were chosen for this study.

 The current systematic review summarizes the fifteen studies included; fourteen^1,5,50-53,20,43-49^ studies indicated that using aloe vera gel along with SRP proved to be advantageous in the treatment of periodontitis, whereas one study^18^ reported no difference between the two groups. Deepu et al^51^ found that aloe vera gel was effective in the short term (one-month follow-up) but had no effect in the long term (two-month and four-month follow-ups). There were no reported side effects from using aloe vera gel in any of these studies. In all these trials, aloe vera was injected into the periodontal pocket after SRP in the test group.

 The primary outcomes assessed in this study were PI, GI, and PPD. This meta-analysis demonstrated that adjunctive use of aloe vera gel with SRP significantly enhanced PI, GI, and PPD in patients with periodontitis. The antiplaque activity of aloe vera can be credited to its antibacterial and antifungal action, especially against *Streptococcus* and *Candida* species.^15,59^ Numerous pharmacologically active substances found in aloe species, including homonataloin, aloeresin, aloe emodin, aloin (the C-glucoside of aloe emodin), and chrysophanol, have all been linked to its antimicrobial activity.^60^ The anti-gingivitis effect of aloe vera can be linked to its antiplaque and potent anti-inflammatory characteristics.^15^ Aloe extracts are effective against bradykinin, histidine, COX-1, and COX-2 enzymes.^60^ Additionally, aloe vera includes elements that aid in forming collagen, including vitamin C, mannose-6-phosphate, dermatan sulfate, and hyaluronic acid, which reduce swelling and gingival bleeding.^15,59^ Aloins can inhibit matrix metalloproteinases (MMPs) because they are structurally analogous to tetracyclines.^61^ Inhibition of MMP-2 and MMP-9 prevents tissue destruction in periodontitis.^62^ Furthermore, aloins inhibit collagenase activity and stimulate cell development, producing greater collagen content.^61^ As a result of these properties, aloe vera improves the PPD.

 The meta-analysis of all three parameters revealed a sizable heterogeneity, which might be attributed to the limitations of clear evidence. Lack of standardization and intra-examiner calibration were noted in most of the studies included in this meta-analysis. Additionally, we encountered inconsistencies in the number of applications and the concentration of aloe vera gel used in these studies. Regrettably, no literature is available on the sustenance and half-life of aloe vera gel when placed in the periodontal pocket. Hence, the substantivity and bio-availability of aloe vera gel cannot be debated. However, Sethi et al^18^ proposed using a bio-adhesive compound with aloe vera gel to keep it in the periodontal pocket for a longer period for greater advantages. The use of aloe vera gel with different concentrations may also have influenced the results of this meta-analysis. Furthermore, as the included studies published their results in different formats, separate analyses were performed for studies with mean and standard deviation data and studies with mean change data. Also, inadequacy in the total number of studies included with language specificity (English) seems to be another drawback.

## Conclusion

 The findings of this study indicate that the adjunctive use of aloe vera gel effectively reduces PI, GI, and PPD in patients with periodontitis, with no adverse effects observed. Based on these results, it is recommended that future research include long-term clinical trials with standardized methodologies to provide more robust evidence on the efficacy of aloe vera gel in periodontal treatment.

## Competing Interests

 The authors declare that they have no competing interests.

## Data Availability Statement

 The study protocol, methodology, search terms used, and statistical analysis plan have been discussed in the manuscript.

## Ethical Approval

 Not applicable.
